# Comparing PET/MRI with PET/CT for Pretreatment Staging of Gastric Cancer

**DOI:** 10.1155/2019/9564627

**Published:** 2019-02-03

**Authors:** Yi Liu, Dong Zheng, Jia-jin Liu, Jian-xin Cui, Hong-qing Xi, Ke-cheng Zhang, Xiao-hui Huang, Bo Wei, Xin-xin Wang, Bai-xuan Xu, Ke Li, Yun-he Gao, Wen-quan Liang, Jia-he Tian, Lin Chen

**Affiliations:** ^1^Department of General Surgery & Institute of General Surgery, Chinese People's Liberation Army General Hospital, Fuxing Road 28, Beijing 100853, China; ^2^Department of Nuclear Medicine, Chinese People's Liberation Army General Hospital, Fuxin Road 28, Beijing 100853, China; ^3^Department of Radiology, Chinese People's Liberation Army 306 Hospital, Beijing 100101, China

## Abstract

18F-FDG PET/MRI has been applied to the diagnosis and preoperative staging in various tumor types; however, reports using PET/MRI in gastric cancer are rare because of motion artifacts. We investigated the value of PET/MRI for preoperative staging compared with PET/CT in gastric cancer (GC). Thirty patients with confirmed GC underwent PET/CT and PET/MRI. TNM staging for each patient was determined from the PET/MRI and PET/CT images. The diagnostic performance of PET/MRI and PET/CT was calculated compared with the pathologic TNM stage. The two methods were compared using statistical analyses. The accuracy for T staging between PET/MRI and PET/CT was 76.9% vs. 57.7%, respectively. In T_1_ and T_4a_ staging, the sensitivity and specificity for PET/MRI vs. PET/CT was 1.0 vs. 0.6 and 1.0 vs. 0.8, respectively. The area under the curve (AUC) for PET/MRI vs. PET/CT was 1.00 vs. 0.78 in the T_1_ stage, 0.73 vs. 0.66 in the T_2_ stage, 0.72 vs. 0.57 in the T_3_ stage, and 0.86 vs. 0.83 in the T_4_ stage. The accuracy for N staging of PET/MRI vs. PET/CT was 53.9% vs. 34.0%, and that for N_0_ vs. N_+_ was 85.0% vs. 77.0%. The sensitivity for PET/MRI in N3 staging was 0.67 and 0 for PET/CT. There was a statistically significant difference in the AUC for N_1_ staging (PET/MRI vs. PET/CT, 0.63 vs. 0.53, *p* = 0.03). SUVmax/ADC positively correlated with tumor volume and Ki-67. PET/MRI performs more accurately in TNM staging compared with PET/CT and is optimal for accurate N staging. SUVmax/ADC has positive correlations with tumor volume and Ki-67.

## 1. Introduction

Although there has been a steady decline in the incidence and mortality rate of gastric cancer (GC) around the world, it remains the fifth most common malignancy and third leading cause of cancer death [1]. GC rates are the highest in Eastern Asia, particularly in China, Korea, Mongolia, and Japan, where the rates are seven times greater than in the USA [2]. Aside from surgery, the only potential curative treatments for GC include neoadjuvant chemotherapy, chemotherapy, and immunotherapy. An accurate pretreatment staging of GC can provide evidence for the correct choice of therapeutic regimen.

Computerized tomography (CT), endoscopic ultrasound (EUS), and positron emission tomography/computed tomography (PET/CT) are routine tools to determine pretreatment staging, all with advantages and limitations. CT remains the first choice for pretreatment staging in GC. Meta-analysis data suggests that the accuracy rate for primary tumor (T) stage identification by CT is 71.5% compared with histology [3]. This rate was lower than when EUS was used, which has been reported as 75% accurate [4]. PET/CT has the highest accuracy among the three examination modalities and additionally plays an important role in detecting occult metastases [5, 6]. Regional tumor (N) staging remains challenging to detect by any of these examinations.

Positron emission tomography/magnetic resonance imaging (PET/MRI) is a promising technique due to the added advantages provided by MRI over CT, including superior soft-tissue contrast, functional imaging, and lack of ionizing radiation [7]. PET/MRI has been applied to more than 16 kinds of oncological diseases, and its diagnostic performance is demonstrated to be similar or better than PET/CT [8]. However, in GC, both MRI and PET/MRI are rarely used for preoperative staging due to respiration and gastric peristalsis during image acquisition, which can lead to motion artifacts. From the limited studies performed to date, the accuracy of MRI in T staging was between 83% and 73.68% [3, 9]. We hypothesized that PET/MRI has a superior performance in GC staging compared with PET/CT or CT; however, the only study comparing PET/MRI to CT did not show any added value of PET/MRI in TN staging [10]. Thus, the aim of this prospective investigation was to assess the value of PET/MRI in GC staging compared with PET/CT.

## 2. Materials and Methods

### 2.1. Patients

**Figure 1:**
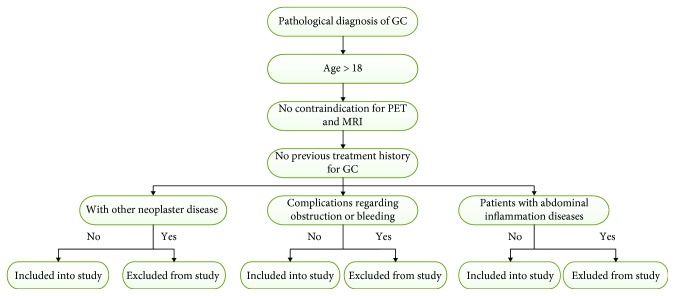
The flowchart of selection criteria.

This prospective study was approved by our institutional review board, and informed consent was signed by all of the recruited patients. We recruited 30 patients that were diagnosed with GC from the Chinese PLA General Hospital, between December 2016 and November 2017. The criteria for inclusion into the study is as follows: pathological diagnosis of GC using endoscopy and biopsy, age greater than 18 years, no previous treatment history for GC, and no contraindications for PET and MRI, such as severe diabetes or a cardiac pacemaker. Exclusion criteria included patients with other neoplastic disease or complications regarding obstructions or bleeding and patients with abdominal inflammatory diseases. The flowchart of selection criteria is shown in Figure 1.

### 2.2. PET/MRI and PET/CT Study

The PET/MRI was performed 3 days after PET/CT. Patients fasted for at least 6 hours and then rested quietly for 20–30 minutes before a body-weight-adapted dose of [18F]-fluorodeoxyglucose (18F-FDG; produced in our institute under good manufacturing practice conditions) at 2.22 to 4.44 MBq (0.08–0.12 mCi)/kg was intravenously injected. A quiet rest of approximately 60 minutes was required before examination. To minimize bowel movement, 10 mg of hyoscine butylbromide (Chengdu No. 1 Drug Research Institute Company Limited, Chengdu, China) was injected intravenously in all patients (no contraindications, such as glaucoma, prostate hypertrophy, or severe heart disease) which was presented 5 minutes prior to PET/MRI examination. Each patient was also asked to drink 1000 mL of water immediately prior to PET/MRI acquisition to distend the stomach and enable better visualization of gastric lesions. Our fully integrated PET/MRI protocol included two parts: (1) a whole-body PET/MRI scan and (2) dedicated stomach MRI. The total acquisition took 30–40 minutes for the whole-body PET/MRI scan and took approximately 20 minutes for the dedicated stomach MRI.

PET/MRI data were acquired by use of an integrated PET/MRI scanner (Biograph mMR, Siemens Healthcare, Erlangen, Germany) that had a YSO crystal-APD PET detector assembly fixed inside a 3.0 T MRI gantry between the body coil and the gradient magnet coil.

All subjects underwent PET/CT scans using the standard protocol used at our institution. Each patient was also asked to drink 1000 mL of water immediately prior to PET/CT acquisition to distend the stomach and enable a better visualization of gastric lesions. Whole-body imaging covered from the chin to the upper thigh with 10–20 min/5 to 7 bed data collection after low-dose CT scanning (120 kV, 100–120 mA/s, 5 mm slice thickness, 5 mm increment, pitch 1) adjusted by the patient's body weight and height, using the scanner (Biograph 64, Siemens Healthcare, Knoxville, TN, USA). PET/CT does not need dedicated stomach scanning, and the absence of it does not affect the diagnostic value of PET/CT and the results of the study. The images were reconstructed with CT attenuation correction (AC) by use of ordered-subset expectation maximization (OSEM) software provided by the venders.

The detailed protocols of the PET/MRI and PET/CT study are supplied in the supplementary data (available here).

### 2.3. Clinical Staging and Gold Standard

Either clinical staging or pathological staging was determined by the 8th edition of the TNM staging system [11]. The detailed imaging criteria of TNM staging are supplied in the supplementary data. The results of postoperative pathological diagnosis served as the reference standard. As the patients in this study had not received a gastrectomy, a combination of clinical features and the results of cytological examination or laparoscopy exploration and imaging examinations of CT and EUS during more than six months of follow-up were regarded as the gold standard for determining the TNM stage.

### 2.4. Image Analysis

Three board-certified nuclear medicine physicians with at least 5 years of experience in PET/CT and 2 years of experience in PET/MRI were responsible for the image analysis in this study. Before the study, the image criteria for GC staging were discussed and determined according to previous studies and our experience [12–14]. The detailed information is given as supplementary data. The reviewers were blinded to the results of the images and pathological reports. Two of the three physicians analyzed the images independently, but for cases where their conclusions were not consistent, the third reviewer judged the divergences and reached a final decision with the other two physicians. The PET/CT and PET/MRI images were analyzed at the end of the study. PET/CT images were analyzed first with cases randomly presented, and 4 weeks later PET/MRI was analyzed to reduce recall bias. The reviewers were asked to determine the T stage as T_1_, T_2,_ T_3_, T_4a_, or T_4b_; the N stage as N_0_, N_1_, N_2_, or N_3_; and the M stage as M_0_ or M_1_.

### 2.5. Statistical Analysis

The primary aim of this study was to investigate the value of PET/MRI in GC staging compared with PET/CT and CT. Diagnostic tests were conducted; using pathological results and clinical information during follow-up as the gold standard, the sensitivity, specificity, and accuracy were calculated. We also performed receiver operating characteristic (ROC) curve analysis for evaluating the diagnostic capability among each imaging platforms in T, N, and M stages. The comparison of sensitivity, specificity, and overall accuracy among the three imaging examinations was calculated with a McNemar test. To test whether the areas under the ROC curves (AUC) were different, the correlation in testing methods was accounted for in the analysis. The correlation between SUVmax/apparent diffusion coefficient (ADC) and clinical features was analyzed by Spearman's rank correlation. A *p* value <0.05 was considered to be a statistically significant difference, and all the statistical analyses were performed using the commercially available MedCalc software program v.12.2.1.0.

## 3. Results

### 3.1. Surgical Operation and Pathologic Staging

Twenty-six patients underwent a curative gastrectomy with D2 lymphadenectomy, and one patient underwent a palliative gastrectomy after preoperative systemic chemotherapy. Four of the 30 patients were diagnosed with distant metastasis: peritoneal seeding (*n* = 1), liver (*n* = 1), retroperitoneal lymph nodes (*n* = 1), and retroperitoneal and left supraclavicular lymph nodes (*n* = 1). The patient with peritoneal seeding was diagnosed by diagnostic laparoscopy and finally confirmed by histological examination after palliative gastrectomy. The other patients with distant metastasis were diagnosed clinically using the PET, CT, and EUS follow-up results. As shown in Table 1, the GC consisted of 21 tubular adenocarcinomas (moderate differentiation in 9 patients and poor differentiation in 12 patients), 2 signet ring cell carcinomas, 5 adenocarcinomas with signet ring cells and mucinous cell components, and 2 adenocarcinomas with neuroendocrine cell components. The male to female ratio was 4 : 1, and the mean age was 58 years (range, 34–76). The average tumor size was 28.57 cm^3^. The pathological T staging of patients who had undergone curative gastrectomy was, respectively, staged as T_1_, T_2_, T_3_, and T_4a_ in 5, 4, 8, and 9 cases, and N staging was N_0_, N_1_, N_2_, and N_3_ in 11, 4, 5, and 6 cases (Table 2).

### 3.2. Diagnostic Performance Analysis

#### 3.2.1. Diagnostic Value of T Staging

**Figure 2:**
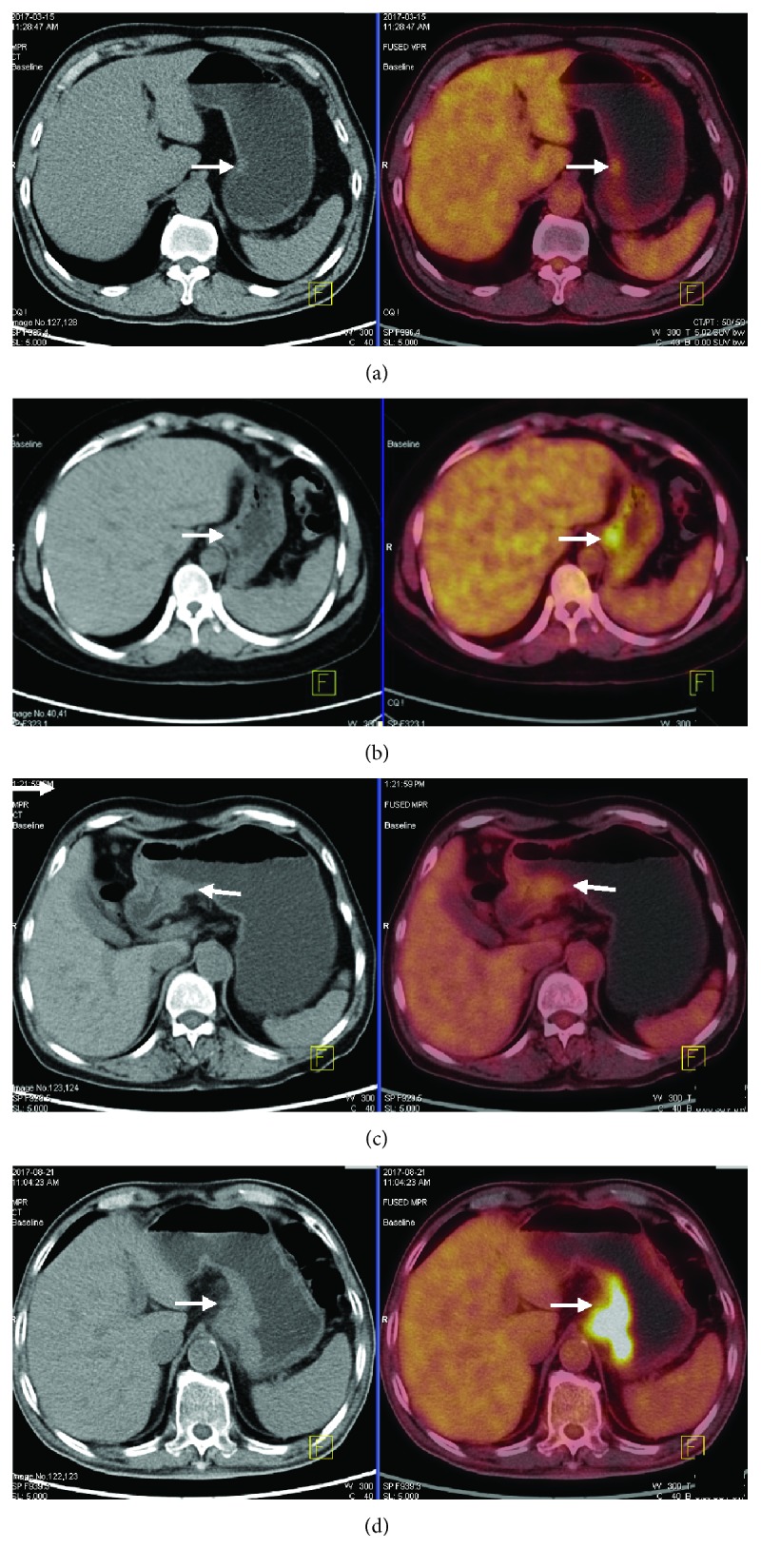
PET/CT images of T staging. (a) A 58-year-old man with gastric carcinoma pathologically diagnosed as stage T1; focal thickening of the gastric wall in the antrum with increased uptake tissues not exceeding the intermediate layer (arrow). (b) A 46-year-old woman with gastric carcinoma pathologically diagnosed as stage T2; focal thickening of the gastric wall in the cardia with increased uptake tissues exceeding the intermediate layer without infiltrating the whole thickened gastric wall (arrow). (c) A 70-year-old man with gastric carcinoma pathologically diagnosed as stage T3; focal thickening of the gastric wall in the antrum, the whole thickened gastric wall is infiltrated by the increased uptake tissues with a smooth and well-defined outer border (arrow). (d) A 74-year-old man with gastric carcinoma pathologically diagnosed as stage T4; thickening of the gastric wall in the cardia and fundus, the whole thickened gastric wall with an irregular outer border is infiltrated by the increased uptake tissues and transmural extension into perigastric fat (arrow).

**Figure 3:**
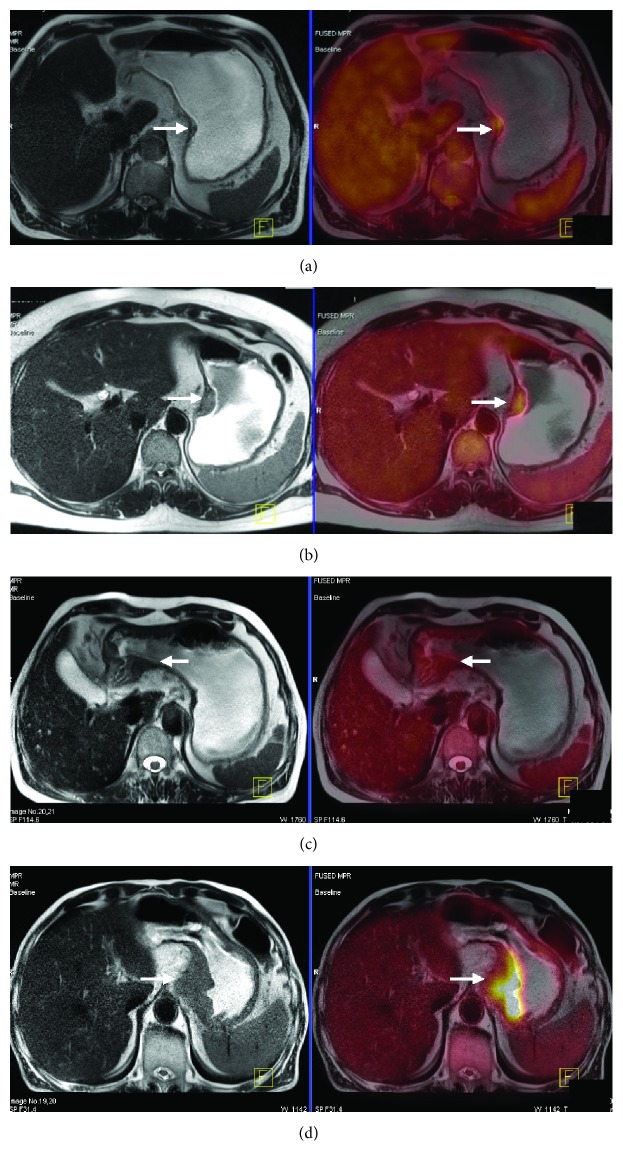
PET/MRI images of T staging. All the patients are corresponding to Figure 1. (a) A 58-year-old man with gastric carcinoma pathologically diagnosed as stage T1. Transverse T2W image shows focal thickening of the gastric wall at the lesser curvature of the stomach, the tumor of low signal intensity confined within the mucosa and submucosa, and the high signal intensity stripe of submucosa appears to be continuous (arrow). (b) A 46-year-old woman with gastric carcinoma pathologically diagnosed as stage T2. Transverse T2W image shows focal thickening of the gastric wall in the cardia with a smooth and well-defined outer border, the tumor of heterogeneous signal intensity involving the muscularis propria, and disruption of the high signal intensity stripe of submucosa (arrow). (c) A 70-year-old man with gastric carcinoma pathologically diagnosed as stage T3. Transverse T2W image shows focal thickening of the gastric wall in the antrum; the whole thickened gastric wall is infiltrated by the low signal intensity of tumor tissues with a smooth and well-defined outer border (arrow). (d) A 74-year-old man with gastric carcinoma pathologically diagnosed as stage T4. Transverse T2W image shows an irregular thickened gastric wall in the cardia and fundus; the whole thickened gastric wall with an irregular outer border is infiltrated by the low signal intensity of tumor tissues and transmural extension into perigastric fat (arrow).

For overall T staging, PET/MRI and PET/CT correctly diagnosed 20 and 15 cases from 26 patients, respectively, and the accuracy of PET/MRI vs. PET/CT was 76.9% vs. 57.7% (*p* = 0.18). The diagnostic sensitivity, specificity, accuracy, and AUC of each stage compared between PET/MRI and PET/CT are summarized in Table 3, and no statistically significant differences were found. In T_1_ and T_4a_ stages, PET/MRI diagnosed all cases correctly (sensitivity = 100%), while PET/MRI and PET/CT had the same specificity regarding the T_4a_ stage (75%). The AUC of PET/MRI vs. PET/CT was 1.00 vs. 0.78 in the T_1_ stage, 0.73 vs. 0.66 in the T_2_ stage, 0.72 vs. 0.57 in the T_3_ stage, and 0.86 vs. 0.83 in the T_4_ stage. PET/MRI or PET/CT performed less well in the T_2_ and T_3_ stages, and PET/CT had a sensitivity of only 25% in the T_3_ stage. The representative cases are shown in Figures 2 and 3 for T staging.

#### 3.2.2. Diagnostic Value of N and M Staging

**Figure 4:**
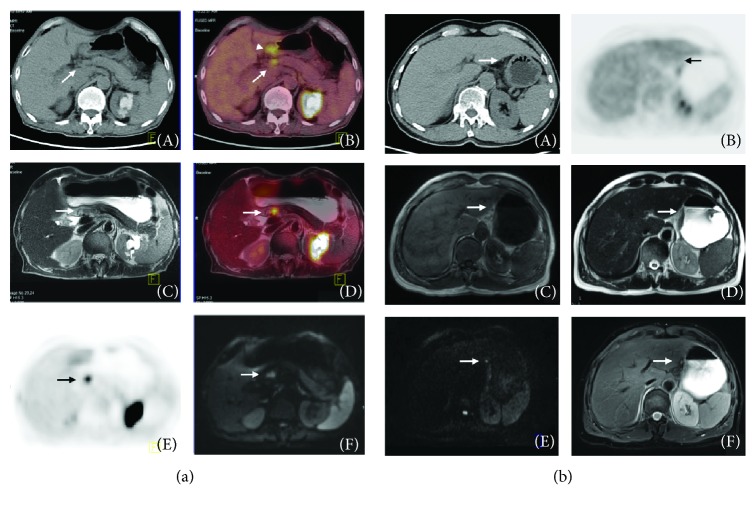
(a) A 75-year-old man with gastric carcinoma diagnosed as lymph node metastasis. (A) The axial CT image shows an enlarged lymph node (arrow) next to the lesion (arrowhead). (B, D) A fusion PET/MRI and PET/CT image shows the enlarged lymph node with FDG uptake and an SUVmax of 3.4 (arrow). (C) One T2-weighted axial image shows a mild-high signal intensity enlarged lymph node (arrow) next to the antrum. (E) One PET image when an obviously avid FDG uptake was observed (arrow). (F) One diffusion-weighted image shows the enlarged lymph node with high signal intensity (arrow), suggesting diffusion restriction. With the aid of these images, indicating a metastatic lymph node, a preoperative diagnosis of N+ could be made. (b) A 67-year-old man with gastric carcinoma diagnosed as a short diameter 5 mm perigastric metastatic lymph node (arrow). The lymph node near the lesser curvature of the stomach is not found as a metastasis lesion on the CT and PET (A, C, D) and no increased FDG uptake (B). It can be diagnosed through diffusion-weighted imaging (*b* = 800) and T2-weighted saturated fat imaging due to its high signal intensity on both modalities (E, F).

The accuracy for PET/MRI and PET/CT for the overall N staging was 53.9% and 34% (*p* = 0.29), but the accuracy for N_0_ vs. N_+_ was 0.82 in PET/MRI and 0.73 in PET/CT. The sensitivity, specificity, and AUC for the N_0_ stage were 0.73, 0.93, and 0.85 in PET/MRI and 0.67, 0.79, and 0.77 in PET/CT, respectively. A statistically significant difference was found in the AUC of PET/MRI vs. PET/CT for the N_1_ stage (0.63 vs. 0.53, *p* = 0.03), although the sensitivity of PET/MRI and PET/CT was the same for the N_1_ and N_2_ staging at 0.25 and 0.2, respectively. For the N_3_ stage, PET/MRI correctly diagnosed 4 cases from 6 patients, but PET/CT diagnosed none of the cases (sensitivity, PET/MRI vs. PET/CT = 0.67 vs. 0).  Figure 4 shows representative images for the detection of positive lymph nodes.

Neither PET/MRI nor PET/CT diagnosed the distant metastasis as peritoneal seeding, but for the other three patients with liver, retroperitoneal lymph nodes, or left supraclavicular lymph nodes, they had similar performance in detecting the lesions.

### 3.3. The Relationship between SUVmax/ADC and Clinical Features

**Figure 5:**
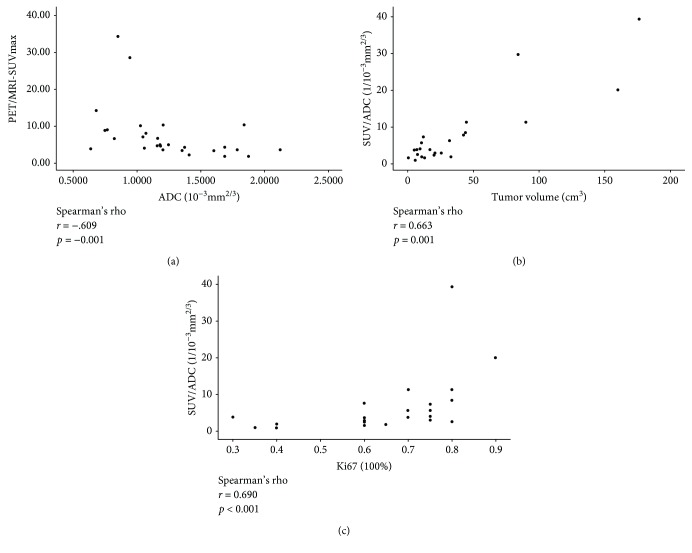
(a) A slight negative correlation was found between PET//MRI-SUVmax and apparent diffusion coefficient (ADC) of the primary lesion. (b, c) Positive correlations exist between SUV/ADC and Ki-67 or the tumor volumes.

As shown in Table 1, the average PET/MRI-SUVmax, PET/CT-SUVmax, and apparent diffusion coefficient (ADC) of all the patients were 7.14, 7.95, and 1.21 × 10^−3^ mm^2/S^. The SUVmax was the lowest in the signet ring cell carcinoma group compared with the other cancer types. A negative correlation was found between the SUVmax and ADC of primary lesions (Spearman's nonparametric correlation, *r* = −0.609, *p* = 0.001). The SUVmax/ADC of primary lesions was found to correlate positively with their size (Spearman's nonparametric correlation, *r* = 0.663, *p* = 0.001) and the proliferation marker, Ki-67 (Spearman's nonparametric correlation, *r* = 0.690, *p* ≤ 0.001). The correlations between these factors are shown in Figure 5. TNM staging, tumor differentiation, and HER-2 status were not found to correlate with the SUVmax/ADC.

## 4. Discussion

^18^F-FDG PTE/MRI is widely accepted as a useful tool in the diagnosis and staging of various types of tumors; however, its application in GC was deemed to be impractical due to motion artifacts caused by respiration or gastric peristalsis and higher examination time cost than PET/CT [15]. Only one study using GC patients has been performed to date and comprises a retrospective comparison between PET/MRI and MDCT [10]. The motion artifacts detected in this study meant that it failed to assess the value of PET/MRI for accurate T and N staging, and any benefit of using PET/MRI in T and N staging compared with MDCT was not determined. Considering that many studies have suggested that MRI performs better than CT in GC [13, 14], we expected that PET/MRI would be more useful in GC staging; thus, we conducted a prospective study comparing PET/MRI with PET/CT for preoperative staging. Before our study, we had tried to overcome the problems associated with examining GC by PET/MRI. To maintain a filled stomach, we tried to continuously inject water into the stomach with a pump system; however, this was unfeasible due to artifacts caused by the pump. Finally, we found that defining the optimal method for stomach imaging and minimizing the examination time is a feasible way to reduce motion artifacts and mismatches between MRI data and PET data (more detail is shown in our protocol). Although no statistical differences were shown in the major test results, the performance of PET/MRI was better than that of PET/CT regarding diagnostic test results and identification of the images.

According to the 7th or 8th edition of the AJCC TNM staging system for GC [16], the T_3_ stage is defined as when the tumor penetrates the subserosal connective tissue without invasion of the visceral peritoneum and is considered the most difficult part of preoperative staging using imaging examinations. The subserosal connective tissue is less than 1/10 of the stomach wall; therefore, it is difficult to identify from the narrow stomach image. PET/MRI can provide high-resolution anatomic data, and it was expected to have better performance than PET/CT in T_3_ staging by our selected imaging sequence. The sensitivity of PET/MRI and PET/CT imaging was 0.50 and 0.25, respectively, and the AUC value was greater for PET/MRI than that for PET/CT. Although neither PET/MRI nor PET/CT achieved an optimal performance in T_3_ staging, PET/MRI still had an advantage compared with PET/CT, even with CT or MRI. Both PET/MRI and PET/CT had a relatively low sensitivity in T_2_ staging, mainly due to the incorrect identification of the T_2_ stage instead of the T_3_ stage. When compared with PET/CT, PET/MRI produced fewer mistakes in the diagnosis of T_2_ or T_3_ patients. PET/MRI correctly identified the tumor staging in the T_1_ and T_4_ stages. The overall accuracy of T staging was 76.9% by PET/MRI and 57.7% by PET/CT. The AC values for CT and MRI, which were reported in a latest review, were 71.5% and 83%, respectively [6]. However, we found that the majority of articles cited in the review were conducted before 2012 and used the 6th edition of the AJCC TNM staging system. The T_3_ stage in the 6th edition is defined as when the tumor invades serosa and is equal to the T_4a_ stage in the 7th edition [17]. It is easier to identify the T stage with the 6th edition than with the 7th edition using imaging examinations. We did not include CT data in our study as patients often had CT examinations in other hospitals, and we could not obtain sufficient image data from these CT measurements. There were 18 patients in our study who underwent CT in our hospital, and the overall accuracy of CT compared with pathological results in T staging was 61%, lower than PET/MRI. From the results of diagnostic tests and comparison of images, we can conclude that PET/MRI performs better than PET/CT for T staging.

Metastatic lymph nodes (LN+) are difficult to detect by imaging techniques as the criteria defining LN+ are mainly size-dependent (raging from >5 mm to >1 cm) [12]. However, LN+ are not always enlarged [18]. In our study, there were 126 LN+ from 768 LN identified by pathological examinations, and the ratio of LN+ <5 mm was 58% (73/126) and the ratio of LN+ with high metabolism in PET was only 9.5% (12/126). Additionally, enlarged LN are not always tumor-positive but can be enlarged due to inflammation [19]. These results show the extreme difficulty in accurate N staging by size-dependent criteria or metabolic criteria. Therefore, most studies that have focused on operative staging only performed a comparison between N_0_ and N+ [10, 20, 21]. In our experience, LN can be identified by diffusion-weighted imaging (DWI) and combined with a T_2_ saturated fat sequence, so PET/MRI can identify more LN and also distinguish more LN+ from them, as shown in Figure 4. PET/MRI using both metabolic criteria and size-dependent criteria and functional imaging has more advantages for N staging than other imaging techniques. The overall accuracy for N staging was 53.85% using PET/MRI and 34% using PET/CT. The overall accuracy of N_0_ vs. N+ was 85% using PET/MRI and 77% using PET/CT, which is better than the latest study of 76.7% using PET/MRI [10] and 64% using EUS. In the N_3_ stage, PET/MRI correctly diagnosed 4 cases from 6 patients while PET/CT diagnosed none. As shown in Table 2, PET/MRI diagnosed 10 cases as the N_3_ stage and PET/CT diagnosed only 1 case. That means PET/MRI has greater power in the diagnosis of LN+ GC. At the same time, less diagnostic cases lead to a lower rate of false-positive identifications; it is one reason why PET/CT achieves better results in GC. These results support the point that PET/MRI is better able to recognize LN+ than is PET/CT and even other imaging examinations.

There are many reasons that impact on the performance of PET/MRI in determining the N stage. First, an incorrect diagnosis of inflammatory LN as positive LN led to an overestimate of N staging. Second, many LN+ cases were diagnosed by PET/MRI but were not identified by pathological examination. Reports suggest that ex vivo lymphadenectomy (EVD) after gastrectomy, which can procure more LN, was more accurately staged than the no EVD group was (*p* < 0.001) with 28% of the no EVD group being inadequately staged [22]. A prospective study that combines EVD with PET/MRI may be needed to assess the true value of PET/MRI in N staging. Here, we conclude that PET/MRI is the optimal imaging technique for N staging as it has the ability to identify a greater number of LN+.

**Figure 6:**
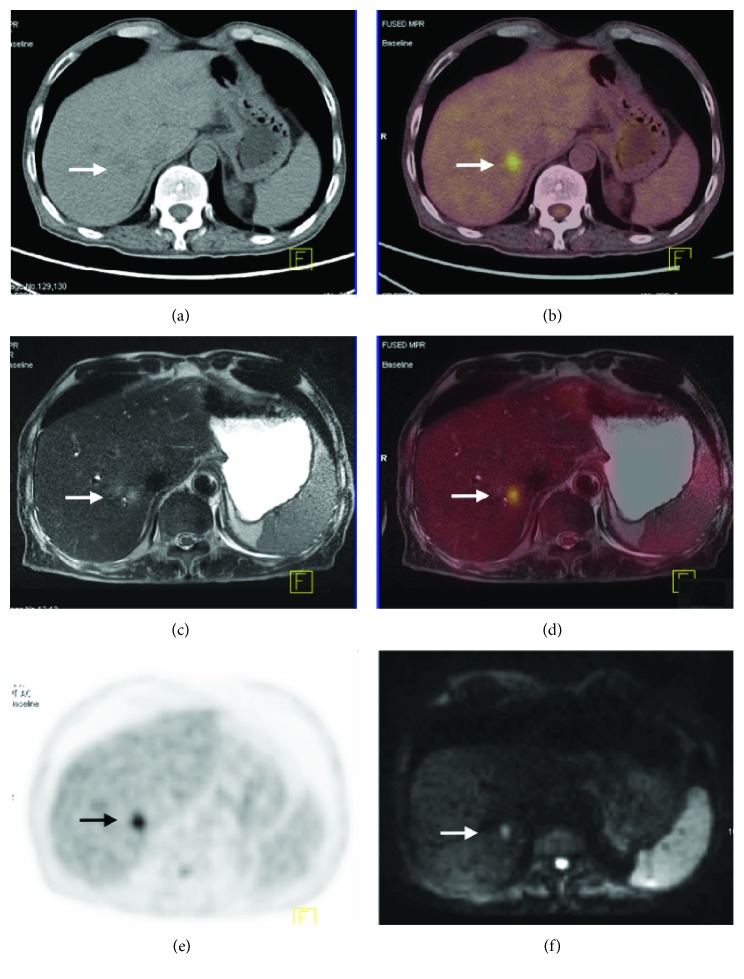
A 75-year-old man with gastric carcinoma diagnosed as liver metastasis proven by clinical information of follow-up. (a) Axial CT image shows suspicious mild low density in segment VII of the liver (arrow). (b, d) On fusion PET/MRI and PET/CT image, the right liver node shows FDG uptake with an SUVmax of 4.3 (arrow). (c) On the T2-weighted axial image, a moderate high signal intensity node is seen (arrow). (e) On the PET image, an obviously avid FDG uptake was observed (arrow). (f) This lesion shows high signal intensity on diffusion-weighted image with a *b*-value of 800 (arrow).

From our data, the performance of PET/MRI is equivalent to PET/CT during M staging for the patient with peritoneal seeding, as both modalities failed to identify the case. Many studies have shown the adequate performance of PET/MRI in M staging, although our data cannot support these conclusions due to the small sample size. The images from the liver metastasis patient suggest that PET/MRI has a significant advantage in diagnosing liver lesions and predicting resectability (as shown in Figure 6). One limitation of this study is the small sample size; we did not have enough patients to identify any differences between the two methods, and we only achieved a statistically significant difference in AUC for N_1_ staging.

Previous studies suggest that SUV in GC is affected by its histological type and tumor volume [23, 24]. The SUV value was lower in small tumors or signet cell carcinomas and is consistent with our results. The use of SUV and ADC to predict the preoperative chemotherapy response has been proven by many studies [25–27]. Here, we investigated the correlations between SUVmax/ADC and clinical characteristics and found that the tumor volume and Ki-67 have a positive correlation with SUVmax/ADC. We hypothesized that the fusion coefficient—SUVmax/ADC of the primary lesion can be a potential predictive marker for preoperative chemotherapy response and long-term survival; however, future work is needed to test this hypothesis.

Nowadays, PET/CT is widely used in the systemic assessment of patients with suspected metastasis. Compared with PET/CT, the application of PET/MRI on GC will not be limited to these patients according to our results. First, PET/MRI has a high diagnostic value on T_1_ and N_0_ staging (Ac: T1, 100%; N0, 85%). For patients with early GC, it can provide more systemic and exact evidence for endoscopic submucosal dissection (ESD) surgery than EUS can. Second, PET/MRI has advantages in N staging; it can recognize more LN+ cases than other examinations. In clinical practice, T staging is primarily used to determine the necessity of neoadjuvant chemotherapy, but with more exact N staging evidence, surgeons may provide patients with more accurate surgical options and chemotherapy. Meanwhile, the use of SUV or ADC to predict the preoperative chemotherapy response is not just a research concept; the Memorial Sloan Kettering Cancer Center has applied it to the treatment of GC [28]. The results of PET/MRI use include many metabolic parameters, such as SUV, ADC, Kep, and Ve. It would be promising for the individualized selection of neoadjuvant chemotherapy. Generally, after the first assessment of GC using CE-CT and EUS, a PET/CT would be conducted if suspected metastatic lesions were identified. In the event of liver metastasis, an MRI would be conducted. The whole process is costly regarding time and money, especially time. PET/MRI can provide both systemic and local evidence for clinical decision-making in 1–2 hours, and the cost of PET/MRI is similar comparable to PET/CT.

## 5. Conclusion

PET/MRI performs better in TN staging compared with PET/CT; it has the potential to be the optimal imaging technique for accurate N staging. Furthermore, we identified that the SUVmax/ADC correlates positively with tumor volume and Ki-67.

## Figures and Tables

**Table 1:** Clinical and tumor characteristics of patients.

Histology	*n*	Male/female	Ages (years)	Size (cm^3^)	SUV_max_-PET/CT	SUV_max_-PET/MRI	ADC_max_ (×10^−3^ mm^2/S^)
Malignant	30	24/6	34-76	28.57 ± 8.08	7.95 ± 1.46	7.14 ± 1.38	1.21 ± 0.45
Tubular adenocarcinoma (h)	0						
Tubular adenocarcinoma (m)	9	8/1	34-64	42.78 ± 19.08	9.02 ± 3.76	8.52 ± 3.39	1.16 ± 0.22
Tubular adenocarcinoma (l)	12	10/2	37-76	9.64 ± 3.72	6.68 ± 1.55	5.05 ± .0.66	1.22 ± 0.10
Signet ring cell carcinoma	2	0/2	47, 62	NA, 6.00	3.31, 1.82	1.51, 1.55	1.70, 1.89
Adenocarcinoma/signet ring cell carcinoma/mucinous adenocarcinoma	5	5/0	48-64	21.33 ± 6.07	6.40 ± 1.05	5.84 ± 1.36	1.22 ± 0.05
Adenocarcinoma/neuroendocrine carcinoma	2	1/1	64, 73	84, 160	24.70, 14.10	28.50, 14.00	0.97 0.70

**Table 2:** Clinical staging and pathological staging.

Stage	Reference standard (*n*, %)	PET/MRI (*n*, %)	PET/CT (*n*, %)
T stage			
1	5 (19.2)	5 (19.2)	4 (15.4)
2	4 (15.4)	3 (11.5)	6 (23.1)
3	8 (30.8)	5 (19.2)	4 (15.4)
4	9 (34.6)	13 (50.0)	12 (46.2)
N stage			
0	11 (42.3)	9 (34.6)	12 (46.2)
1	4 (15.4)	1 (3.8)	5 (19.2)
2	5 (19.2)	6 (23.1)	8 (30.8)
3	6 (23.1)	10 (38.5)	1 (3.8)
M1	4	3	3

**Table 3:** Diagnostic results of PET/MRI versus; PET/CT according to the gold standard.

		Se (95% confidence)	Sp (95% confidence)	AUC (95% confidence)	Ac (%)
T1	PET/MRI	1.00 (0.46-1.00)	1.00 (0.81-1.00)	1.00 (0.87-1.00)	1.00
	PET/CT	0.60 (0.17-0.93)	0.95 (0.74-1.00)	0.78 (0.57-0.92)	0.88
T2	PET/MRI	0.50 (0.09-0.91)	0.95 (0.75-1.00)	0.73 (0.52-0.88)	0.88
	PET/CT	0.50 (0.09-0.91)	0.82 (0.59-0.94)	0.66 (0.45-0.83)	0.77
T3	PET/MRI	0.50 (0.17-0.83)	0.94 (0.71-1.00)	0.72 (0.51-0.88)	0.81
	PET/CT	0.25 (0.04-0.64)	0.89 (0.63-0.98)	0.57 (0.34-0.74)	0.69
T4	PET/MRI	1.00 (0.63-1.00)	0.75 (0.47-0.92)	0.88 (0.70-0.98)	0.81
	PET/CT	0.89 (0.51-0.99)	0.75 (0.47-0.92)	0.83 (0.63-0.94)	0.77
N0	PET/MRI	0.73 (0.39-0.93)	0.93 (0.66-1.00)	0.83 (0.63-0.95)	0.85
	PET/CT	0.73 (0.39-0.93)	0.73 (0.45-0.91)	0.73 (0.52-0.88)	0.73
N1	PET/MRI	0.25 (0.01-0.78)	1.00 (0.82-1.00)	0.63^a^ (0.52-0.81)	0.88
	PET/CT	0.25 (0.01-0.78)	0.82 (0.59-0.94)	0.53 (0.33-0.73)	0.73
N2	PET/MRI	0.20 (0.01-0.70)	0.76 (0.52-0.91)	0.52 (0.32-0.72)	0.65
	PET/CT	0.20 (0.01-0.70)	0.67 (0.43-0.85)	0.57 (0.36-0.76)	0.58
N3	PET/MRI	0.67 (0.24-0.94)	0.70 (0.46-0.87)	0.68 (0.47-0.85)	0.69
	PET/CT	0 (0-0.44)	0.95 (0.73-1.00)	0.53 (0.32-0.72)	0.73

^a^The *p* value was 0.03 (PET/MRI vs. PET/CT).

## Data Availability

The pathology and imaging data used to support the findings of this study are restricted by the Chinese People's Liberation Army General Hospital ethics review board in order to protect patients' privacy. Data are available from Yi Liu (E-mail: 18810751766@163.com) for researchers who meet the criteria for access to confidential data.

## Supplementary Materials

Supplementary Materials The supplementary material mainly included three parts. Firstly, the detail TNM staging of each patient using imaging examinations and pathology diagnosis; secondly, the operation process of PET/CT and PET/MRI and the detail information of sequences which we had applied into PET/MRI; and thirdly, the imaging criteria of TNM staging. The information may be helpful to the reviewers but not necessary.
