# Supplementary material for: Comparing PET/MRI with PET/CT for Pretreatment Staging of Gastric Cancer
Source: Gastroenterol Res Pract. 2019 Feb 3;2019:9564627. doi: 10.1155/2019/9564627 (PMC6378050; doi:10.1155/2019/9564627)
Supplement: Supplementary Materials — The supplementary material mainly included three parts. Firstly, the detail TNM staging of each patient using imaging examinations and pathology diagnosis; secondly, the operation process of PET/CT and PET/MRI and the detail information of sequences which we had applied into PET/MRI; and thirdly, the imaging criteria of TNM staging. The information may be helpful to the reviewers but not necessary. [file 9564627.f1.docx]

**SUPPELEMETARY DATA**

**TNM staging of patients**

| **ID** | **Sex** | **Age** | **PET/MRI** | **PET/CT** | **CT** | **pTNM** |
| --- | --- | --- | --- | --- | --- | --- |
| Y24800xx | Female | 44 | T4N3M1 | T4N2M1 | None | None |
| Y25421xx | Male | 63 | T4N3M1 | T4N3M1 | None | None |
| Y27705xx | Male | 74 | T4N2M1 | T4N2M1 | None | None |
| Y24455xx | Male | 37 | T4N1M1 | T4N1M1 | T4N1M1 | T4aN1M1 |
| Y30091xx | Female | 47 | T1N0M0 | T2N0M0 | T1N0M0 | T1N0M0 |
| Y26073xx | Male | 58 | T1N0M0 | T2N0M0 | T1N0M0 | T1N0M0 |
| K01291xx | Male | 53 | T1N0M0 | T1N0M0 | T3N1M0 | T1N0M0 |
| 5998xx | Male | 63 | T1N0M0 | T1N0M0 | T1N0M0 | T1N0M0 |
| Y27271xx | Male | 62 | T1N0M0 | T1N0M0 | T1N0M0 | T1N0M0 |
| Y27556xx | Male | 64 | T4N3M0 | T4N2M0 | T4N1M0 | T2N1M0 |
| Y27684xx | Female | 45 | T2N2M0 | T2N0M0 | None | T2N1M0 |
| K00183xx | Male | 63 | T3N2M0 | T3N1M0 | T3N1M0 | T2N2M0 |
| A3297xx | Male | 48 | T2N3M0 | T2N0M0 | T2N0M0 | T2N3M0 |
| Y28966xx | Female | 64 | T4aN3M0 | T3N1M0 | T4N0M0 | T3N0M0 |
| G1020xx | Female | 62 | T2N0M0 | T1N0M0 | None | T3N0M0 |
| K0119339 | Male | 62 | T3N0M0 | T2N0M0 | T3N0M0 | T3N0M0 |
| K01293xx | Male | 50 | T4aN3M0 | T3N1M0 | T4N1M0 | T3N2M0 |
| Y30621xx | Male | 60 | T4aN3M0 | T4aN2M0 | None | T3N2M0 |
| Y28658xx | Female | 70 | T3N2M0 | T4N2M0 | None | T3N3M0 |
| Y24797xx | Male | 67 | T3N1M0 | T2N0M0 | T2N0M0 | T3N3M0 |
| G1029xx | Male | 34 | T3N3M0 | T4N2M0 | None | T3N3M0 |
| Y2458933 | Male | 62 | T4aN1M0 | T4N1M0 | T4N1M0 | T4aN1M0 |
| Y26572xx | Male | 64 | T4N0M0 | T3N0M0 | None | T4aN2M0 |
| Y29637xx | Male | 60 | T4aN3M0 | T4N2M0 | T4aN3M0 | T4aN3M0 |
| Y29665xx | Male | 51 | T4aN3M0 | T4N1M0 | T4N2M0 | T4bN3M0 |
| Y28347xx | Male | 43 | T4aN3M0 | T4N2M0 | T4N1M0 | T4N0M0 |
| Y29241xx | Male | 61 | T4aN2M0 | T4N0M0 | None | T4N0M0 |
| K00194xx | Male | 76 | T4aN0M0 | T4N2M0 | T2N2M0 | T4N0M0 |
| K01148xx | Male | 73 | T4aN2M0 | T4N2M0 | T4N2M0 | T4N1M0 |
| Y2604372 | Male | 64 | T4N3M0 | T4N3M0 | None | T4N2M0 |

**PET/CT** **techniques**

All subjects underwent PET/CT scans using 18F-fluorodeoxyglucose (18F-FDG; produced in our institute under good manufacturing practice conditions) within 2 weeks before surgery with the protocol of PET/CT scanning used at our institution. Before imaging, patients fasted for 6 h and rested for at least 20 min in a quiet waiting room before intravenous administration of 18F-FDG at 2.22 to 4.44 MBq (0.08–0.12 mCi)/kg. Patients were asked to continue their comfortable resting position for another 55 to 60 min. Each patient was also asked to drink 1000 mL of water immediately prior to PET/CT acquisition in order to distend the stomach and enable a better visualization of gastric lesions. Whole-body imaging covered from the chin to upper thigh with 10 to 20 min/5 to 7 bed data collection after low-dose CT scanning (120 kV, 100–120 mA/s, 5mm slice thickness, 5mm increment, pitch 1) adjusted by the patient's body weight and height, using the scanner (Biograph 64, Siemens Healthcare, Knoxville, TN, USA). The images were reconstructed with CT attenuation correction (AC) by use of Ordered Subsets Expectation Maximization (OSEM) software provided by the venders.

**PET/MRI techniques**

The PET/MRI was performed three days after PET/CT. PET/MRI data were acquired by use of an integrated PET/MRI scanner (Biograph mMR, Siemens Healthcare, Erlangen, Germany) that had a YSO crystal-APD PET detector assembly fixed inside a 3.0T MRI gantry between the body coil and gradient magnet coil. All patients fasted for at least 6 hours before the PET/MRI examination. Serum glucose levels were checked with a blood glucose meter prior to the injection and were less than 200 mg/dl (11.1mmol/L) in all patients. A body-weight-adapted dose of 18F-FDG (2.22 to 4.44 MBq/kg) was intravenously injected and PET/MRI was taken approximately 60 minutes (mean, 60 ± 8 minutes) after injection.

For the first part of the examination for whole-body PET/MRI, PET/MRI scanning was done from the level of the thigh to the brain vertex. To minimize bowel movement, 10 mg of hyoscine butylbromide (Chengdu NO. 1 Drug Research Institute Company Limited, Chengdu, China) was injected intravenously in all patients (no contraindications，such as glaucoma, prostate hypertrophy or severe heart disease) were presented 5 minutes prior to PET/MRI examination. Each patient was also asked to drink 1000 mL of water immediately prior to PET/MRI acquisition in order to distend the stomach and enable a better visualization of gastric lesions. The axial range of each bed position was 25.8 cm long with a 6.1 cm overlap between adjacent bed positions, and therefore, in order to obtain coverage from the brain to the mid-thigh, 5 bed positions were required. Combined MRI sequences included transaxial 3-D volumetric interpolated breath-hold T1-weighted sequence (T1 3D-VIBE), transaxial T2-weighted sequence turbo spin echo with fat saturation (T2 TSE-FS), transaxial diffusion-weighted image (DWI) sequences with double b values (50 and 800 s/mm2) acquired after a coronal fast-view T1-weighted localizer sequence and a transaxial 2-point Dixon sequence to generate an MRI-based AC map. Both MRI and PET mages were acquired simultaneously at 5 min per bed position (BP), and the total acquisition took 30∼40 min over 5∼7 BP for each body scan.

After the acquisition of whole-body PET/MRI, dedicated stomach protocol MR examination was done. In our protocol, high resolution half-Fourier acquisition, single-shot, turbo spine-echo (HASTE) were acquired using the following parameters: relaxation time (TR) = 2060 msec, echo time (TE) = 83 msec, slice thickness = 3 mm, number of excitations (NEX) = 3; field of view (FOV) = 23.6 x 32.0 cm^2^, matrix = 378 x512, and number of slices = 40 , T1-weighted volumetric interpolated breath-hold examination (VIBE) images were acquired with three different flip angles using the following parameters: relaxation time (TR) = 4.04 msec, echo time (TE) = 1.24 msec, flip angles (a) = 3, 15, and 35, slice thickness = 3 mm, number of excitations (NEX) = 3, field of view (FOV) = 32.8 x 42.0 cm^2^, matrix = 374 x 640, and number of slices = 64. All patients were instructed to breathe as quietly as possible during the examination. We also performed diffusion-weighted imaging (DWI) using a single-shot echo-planar imaging sequence using b values of 0, 200, 800. The apparent diffusion coefficient (ADC) map was made from DWI using different b values. The scan time for dedicated stomach MR imaging is approximately 20 minutes. Therefore, a 50-60 minutes scan time was needed for our PET/MRI protocol.

**Imaging analysis of TNM staging**

18F-FDG PET/CT images were evaluated visually and semi-quantitatively in three planes (trans-axial, coronal and sagittal). A lesion was determined to be cancerous when the gastric wall showed a focal thickening of at least 5 mm or greater and FDG uptake in the thickened gastric wall was greater than that of the adjacent gastric wall. The imaging criteria for each T stage were: T1 stage(tumors confined to the submucosal layer), suspicious focal thickening of the gastric wall with increased uptake tissues not exceeding the intermediate layer; T2 stage (tumors invading the proper muscle layer), thickening of the gastric wall with increased uptake tissues exceeding the intermediate layer without infiltrating the whole thickened gastric wall; T3 stage (tumors penetrating the subserosal connective tissue without invasion of the visceral peritoneum or adjacent structures), the whole thickened gastric wall is infiltrated by the increased uptake tissues with smooth and well-defined outer border; T4 stage (tumors invading the visceral peritoneum or even adjacent organs); the whole thickened gastric wall with irregular outer border is infiltrated by the increased uptake tissues and transmural extension into perigastric fat or stomach‑adjacent organs.

As for the analysis of 18F-FDG PET/MR images, except methods mentioned above, high-resolution T2-weighted imaging is the key sequence for the T-staging of gastric cancer. On T2-weighted MRI, the gastric wall mucosa shows low signal intensity, the submucosa shows high signal intensity, the muscularis propria shows low signal intensity, the perigastric fat shows high signal intensity, based on this gastric wall layer differentiation on T2-weighted imaging, disruption of the layer and focal altered signal areas within the layer by the tumor were considered as the sign of invasion so that the T-staging of gastric cancer can be made.

Lymph nodes were considered metastatic if they were large than 5 mm in the short-axis diameter and oval or higher FDG uptake than normal tissues. N1: positive sign in 1–2 regional nodes, N2: positive sign in 3–6 regional nodes, N3: positive sign in 7 or more regional nodes. Additional significantly higher abnormal FDG uptake compared to the uptake of the surrounding tissue in the body was documented for the presence of distant metastases.
